# Mesoscopic Heterogeneous Modeling Method for Polyurethane-Solidified Ballast Bed Based on Virtual Ray Casting Algorithm

**DOI:** 10.3390/ma19030474

**Published:** 2026-01-24

**Authors:** Yang Xu, Zhaochuan Sheng, Jingyu Zhang, Hongyang Han, Xing Ling, Xu Zhang, Luchao Qie

**Affiliations:** 1 Railway Engineering Research Institute, China Academy of Railway Sciences Corporation Limited, Beijing 100081, China; xuyangcars@126.com (Y.X.); cz_jingyuz@163.com (J.Z.); hhycars@163.com (H.H.); qlcballast@126.com (L.Q.); 2 State Key Laboratory for Track System of High-Speed Railway, Beijing 100081, China; 3 School of Mechanics and Civil Engineering, China University of Mining and Technology, Beijing 100083, China; szc010708@163.com; 4 School of Civil and Transportation Engineering, Guangdong University of Technology, Guangzhou 510006, China; xuzhang@gdut.edu.cn

**Keywords:** polyurethane-solidified ballast bed, mesoscopic modeling, composite heterogeneous materials, virtual ray casting, sleeper width optimization, Discrete Element Method, Finite Element Method

## Abstract

**Highlights:**

**What are the main findings?**

- A finite element method and virtual projection-based mesoscale modeling approach is proposed to replace X-ray computed tomography (XCT), thereby addressing XCT’s constraints of limited specimen size and high equipment cost.
- The optimal finite-element mesh size is confirmed, ensuring high ballast volume fidelity, consistency with compression tests, and balancing stability accuracy and computational efficiency.
- A suitable sleeper width is recommended, acting as the threshold for reduced displacement sensitivity, ensuring optimal stability and supporting sleeper–ballast collaborative design.
- Polyurethane-solidified ballast beds’ meso-mechanism is revealed: ballast stress concentrates at particle contacts, polyurethane buffers loads, and insufficient polyurethane thickness risks local stress concentration.

**What are the implications of the main findings?**

- The proposed method has cross-material versatility, extendable to other multi-material composites, reducing research costs and highlighting long-term economic value.

**Abstract:**

This study introduces a mesoscale modeling methodology for polyurethane-solidified ballast beds (PSBBs) that eliminates reliance on X-ray computed tomography (XCT) and addresses constraints in specimen size, capital cost, and post-processing complexity. The approach couples the Discrete Element Method (DEM) with the Finite Element Method (FEM). A high-fidelity discrete-element geometry is reconstructed from three-dimensional laser scans of ballast particles. The virtual-ray casting algorithm is then employed to identify the spatial distribution of ballast and polyurethane and map this information onto the finite-element mesh, enabling heterogeneous material reconstruction at the mesoscale. The accuracy of the model and mesh convergence are validated through comparisons with laboratory uniaxial compression tests, determining the optimal mesh size to be 0.4 times the minimum particle size (0.4 *D*_min_). Based on this, a parametric study on the effect of sleeper width on ballast bed mechanical responses is conducted, revealing that when the sleeper width is no less than 0.73 times the ballast bed width (0.73 *W*_b_) an optimal balance between stress diffusion and displacement control is achieved. This method demonstrates excellent cross-material applicability and can be extended to mesoscale modeling and performance evaluation of other multiphase particle–binder composite systems.

## 1. Introduction

Ballasted trackbeds are the primary load-bearing layer of railway infrastructure. They transfer sleeper loads and diffuse stresses into the subgrade. Their stability directly controls track geometry, operational safety, and maintenance costs [1,2]. Because construction is straightforward and costs are controllable, ballasted track remains widely used. However, granular ballast is prone to sliding, abrasion, and attrition, which destabilize the bed and degrade surface quality, thereby requiring frequent maintenance [3]. To slow deterioration and reduce life-cycle costs, polyurethane-solidified ballast has gained prominence. Polyurethane mixtures are injected into intergranular voids and, through percolation–foaming–curing, create a bonded aggregate skeleton. This skeleton combines the elasticity of ballasted track with the monolithic integrity and low-maintenance characteristics of slab systems [2]. Two implementation routes are common. (i) Rigid consolidation (e.g., XiTRACK), where non-foaming polyurethane bonds grain contacts and markedly reduces settlement under cyclic loading [4]. (ii) Pore filling, where foamed polyurethane occupies voids to block fouling ingress while preserving bed resilience; resilient polyurethane-solidified ballast thereby balances deformation resistance and energy dissipation [5].

Although field trials and laboratory tests show that polyurethane-solidified ballast limits settlement and lowers maintenance demand, engineering-ready quantitative metrics and codified mesoscale design criteria remain scarce. In particular, metrics that characterize load diffusion and cooperative load sharing between the granular skeleton and the polyurethane infill are not yet standardized. Guidance that bridges mesoscale mechanisms to system-level performance as a function of geometric parameters (e.g., sleeper width) is also missing. Addressing this gap requires practicable mesoscale numerical modeling and mechanics-based analysis.

Most studies evaluate global structural response under an assumption of macroscale homogeneity. The finite element method (FEM) is a mature and efficient workflow widely used for track design and parameter sensitivity analyses [6,7,8,9]. However, homogenized models cannot faithfully represent the heterogeneous ballast–polyurethane composite, nor the particle-shape-induced local stress concentrations and phase connectivity that govern load transfer and damage risk.

Meanwhile, the Discrete Element Method provides a natural framework for contact interactions in granular media and has been extensively used to investigate ballast behavior and reinforcement effects [10,11,12,13,14], including the influence of polyurethane infusion on ballast-bed deformation resistance [15,16,17]. In addition, the Discrete Element Method literature has reported the influences of particle morphology, reinforcement measures, vehicle–track coupling, interface behavior, and damage-related processes on ballast response [18,19,20,21,22,23,24,25]. Nevertheless, in polyurethane-solidified ballast, conventional Discrete Element Method implementations commonly approximate the polymer phase via idealized contact-bond models—appropriate for contact-point consolidation [26,27,28] but insufficient to resolve the volume-connected pore-filling configuration and its buffering role in load transfer. These limitations motivate a coupled methodology that combines particle-resolved geometry with continuum-scale solvers, while enabling mesh-consistent phase discrimination for heterogeneous composites.

High-fidelity observation and reconstruction of mesostructure are widely achieved using X-ray computed tomography, which has played an important role in concrete and related composite systems for quantifying pore networks, aggregate distributions, and linking microstructure to macroscopic properties [29,30,31,32,33,34,35]. Representative studies have further integrated tomography with full-field measurement techniques (e.g., digital image correlation/digital volume correlation) to investigate strain localization, crack propagation, and damage evolution across composite variants [36,37,38,39]. Building on tomography data, image-based mesoscale modeling strategies—including segmentation-driven finite-element reconstruction and mapping schemes that embed spatial heterogeneity into continuum calculations—have been developed and shown capable of reproducing experimentally observed patterns [40,41,42,43].

Overall, XCT has enabled geometry-faithful, mechanics-coupled mesoscale modeling, but the approach carries notable limitations. First, specimen size is constrained by hardware and image quality: to maintain scanning fidelity, diameters generally must be limited to ≤100 mm [44], whereas engineering-scale polyurethane-solidified ballast specimens far exceed the practical XCT envelope, precluding mesostructural characterization at relevant scales. Second, capital and operating costs are high; statistically meaningful controlled studies typically require large sample counts, necessitating repeated specimen fabrication and scanning—thereby depressing cost-efficiency and imposing non-trivial environmental burdens. These constraints motivate the exploration of virtualized alternatives for geometric identification and mesh-consistent phase mapping, beyond the traditional CT-centric pipeline.

A consolidated summary of the foregoing methods is presented in Table 1.

**Figure 1:**
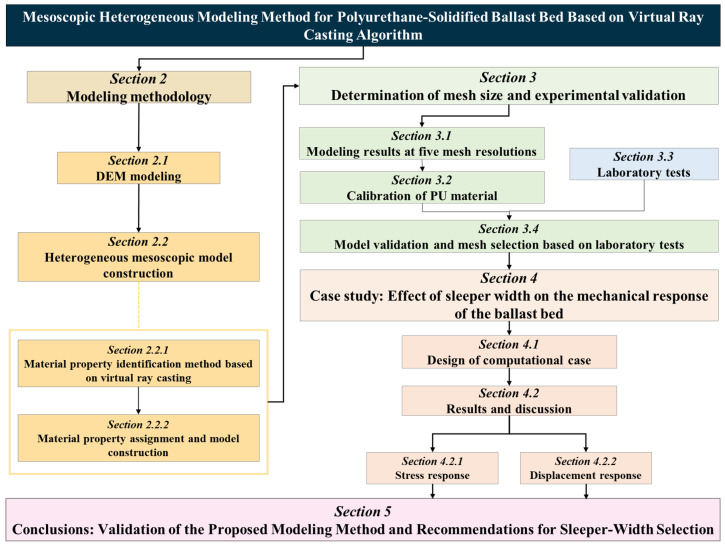
Overall conceptual flowchart of the mesoscopic modeling methodology for polyurethane-solidified ballast beds based on virtual ray casting.

**Figure 2:**
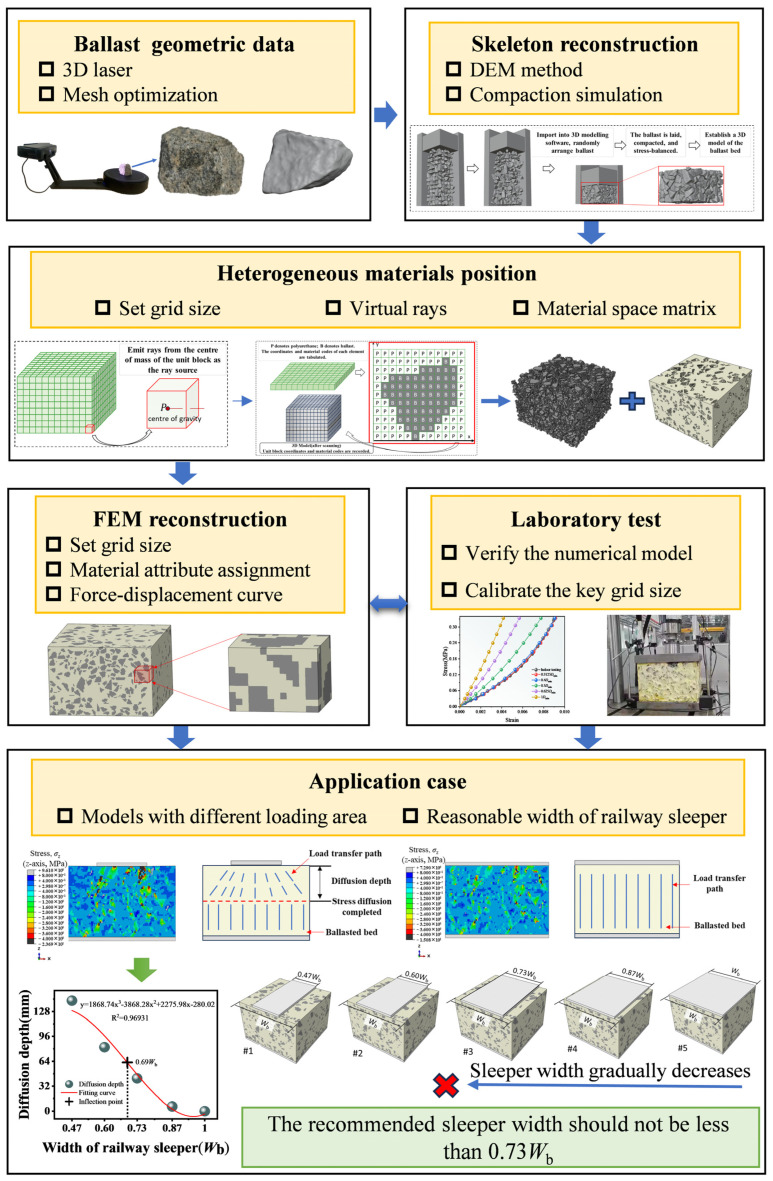
Stepwise technical roadmap from 3D scanning to FEM reconstruction and sleeper width analysis.

To address the foregoing limitations, an image-free, mesostructure-resolved workflow is introduced that tightly couples virtual ray casting with the Discrete Element Method (DEM) and the Finite Element Method (FEM). Virtual rays perform element-scale phase discrimination and volume-fraction mapping on particle-resolved geometries, thereby supplanting X-ray computed tomography (XCT) for large specimens and circumventing its specimen-size constraint, capital cost, and labor-intensive processing. The overall conceptual flowchart is shown in Figure 1. Within this framework, a mesostructural model of polyurethane-solidified ballast is constructed, validated against laboratory tests, and employed to calibrate key mesh-resolution parameters. Feasibility is demonstrated via a case study quantifying the influence of sleeper width on mechanical response. Beyond this exemplar, the workflow generalizes to other large-scale, heterogeneous granular–binder composites, enabling mesostructure-grounded modelling and parametric evaluation at engineering scale. The stepwise technical roadmap from 3D scanning to FEM reconstruction and sleeper width analysis is summarized in Figure 2.

## 2. Modelling of Heterogeneous Materials Based on DEM and Virtual Projection Algorithm

### 2.1. Establishment of Discrete Element Model

**Figure 3:**
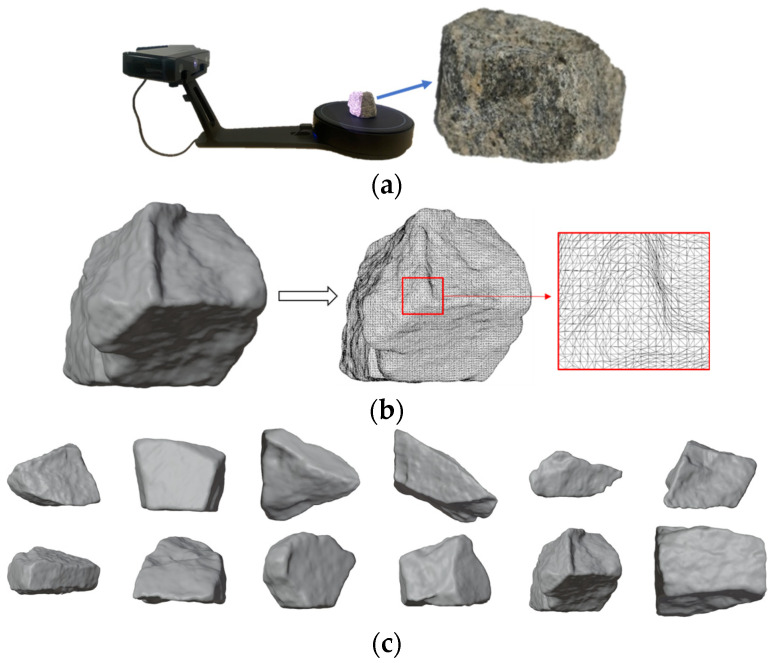
Ballast particle morphology obtained by three-dimensional laser scanning; (**a**) Laser scanning of real ballast particles; (**b**) Discrete element model of a single ballast particle; (**c**) Numerical models of different ballast particles.

The establishment of the Discrete Element Model takes the accurate reproduction of the geometric morphology and spatial arrangement characteristics of ballast particles as its core goal. With reference to previous studies [45,46,47,48], the real surface morphology of natural ballast particles was collected using 3D laser scanning technology (Figure 3a), and the maximum triangular meshing processing was adopted to retain micro-geometric features such as particle edges and textures, thereby ensuring morphological fidelity (Figure 3b). Based on the scanning data, more than 500 ballast particle samples covering different particle size and shapes were established, which meet the requirement for characterizing the diversity of ballast particles. The 3D models of typical ballast particles are shown in Figure 3c. It should be noted that the particles illustrated in Figure 3c can be scaled proportionally to meet the gradation requirements; thus, the particles in the figure are presented solely for demonstrating particle morphology, with no comparison of particle sizes intended.

In accordance with previous studies [26], the gradation of ballast complies with the requirements specified in Table 2, and the discrete element model (DEM) was generated within a rigid box of 300 mm × 300 mm × 300 mm.

**Figure 4:**
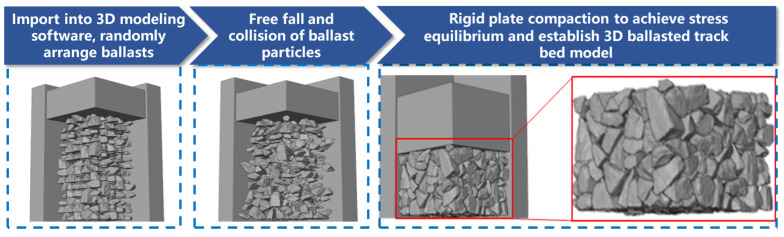
Generation process of the three-dimensional discrete element model of the ballast bed.

All DEM ballast particles are rigid bodies. The random placement and compaction of ballast particles were achieved via a self-developed random placement program, and the DEM contact mechanics algorithm was used to simulate the processes of particle falling, and compaction. The compacted DEM completely retains the irregular morphology of ballast particles, the contact relationships between particles, and the pore distribution characteristics, providing a reliable geometric benchmark for subsequent material identification and mechanical analysis. The generation process is shown in Figure 4.

### 2.2. Construction of a Heterogeneous Mesoscopic Model Based on the Virtual Ray Casting Method

#### 2.2.1. Material Identification Based on Virtual Ray Casting

**Figure 5:**
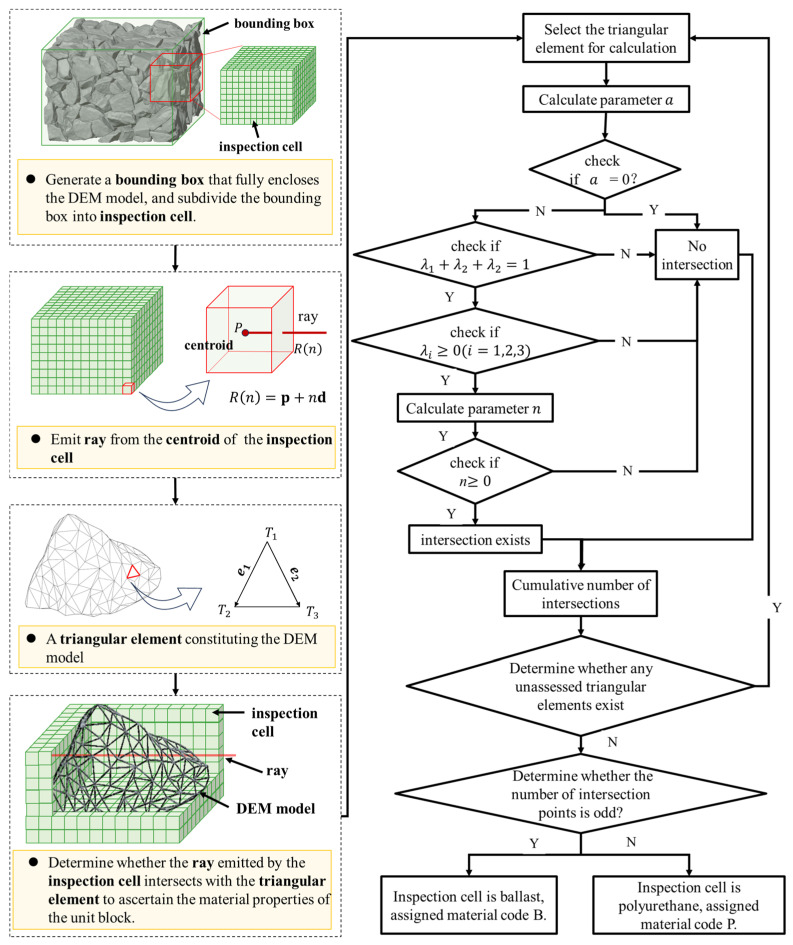
Flowchart of the scanning procedure based on the virtual ray casting method.

Numerous mesoscale modeling methods have been developed for concrete. Table 3 summarizes several representative approaches, along with their modeling approach, advantage, disadvantage and highlight. Building upon these foundations and inspired by the principles of X-ray computed tomography (XCT), this study proposes a novel mesoscale modeling strategy. It introduces a virtual ray-casting method to achieve a clear separation between the aggregate and polyurethane phases in the computational model. Based on a ballast-bed geometry resolved by the Discrete Element Method (DEM), the strategy enables fast, high-accuracy, element-wise material identification, thereby providing a rigorous geometric foundation for subsequent material assignment on the Finite Element Method (FEM) mesh. The procedure is shown in Figure 5, with the detailed steps as follows:

(1) Bounding-box discretization

First, the three-dimensional ballast model generated with the discrete element method is imported into the FEM code. A rectangular cuboid bounding box that fully encloses the geometry is created and discretized into uniformly sized cubic inspection cells. The size of these inspection cells is set identical to the mesh size to be used later in the finite element model, ensuring a one-to-one correspondence between the material classification results and the numerical computation grid.

Compared with the digital-image approach of [44], which requires re-fabricating specimens and performing X-ray computed tomography scanning, the present method discretizes the computational domain by means of a virtual bounding box and therefore does not rely on physical experiments. This not only avoids the high cost and cumbersome procedures of conventional methods but also allows the inspection cell size to be flexibly adjusted to modeling needs, thereby markedly improving modeling efficiency.

(2) Parallelism check between the ray and triangular elements

Using the centroid of each inspection cell as the ray origin, a virtual ray is emitted along a fixed direction, e.g., the positive Z-axis. Because the surface of the ballast model is tessellated by triangular elements, the material type of an inspection cell is determined by the parity of the total number of ray–triangle intersections. An odd count indicates that the cell centroid lies inside a ballast particle and is classified as ballast, whereas an even count indicates that it lies in the polyurethane region and is classified as polyurethane. The procedure is as follows:

Let the position vector of the ray origin (the inspection-cell centroid P) be *p*, and write the ray as $R\left(n\right)=p+nd$ (where *d* is the ray direction vector and *n* is the ray parameter). For an arbitrary surface triangular element with vertices $∆T_{1}T_{2}T_{3}$, the position vectors of its vertices are $t_{1}$, $t_{2}$, and $t_{3}$, respectively, and the edge vectors are defined as $e_{1}=t_{2}-t_{1}$ and $e_{2}=t_{3}-t_{1}$. The cross product $h_{1}$ can be computed using Equation (1) where $h_{1}$ is perpendicular to both $e_{2}$ and d, and the test parameter a can be evaluated according to Equation (2).

(1) $$h_{1}=d\times e_{2}$$

(2) $$a=e_{1}\times h_{1}$$

If a=0, then $e_{1}$ is perpendicular to $h_{1}$, indicating that the ray is parallel to the plane of the triangular element and there is no valid intersection. if a≠0, the ray or its extension in the opposite direction has a unique intersection with the plane of the triangular element, and the procedure proceeds to the next step.

(3) Determination of whether the intersection lies inside the triangular element

Let the intersection of the ray with the plane of the triangular element be $P^{\prime }$, and its position vector $p^{\prime }$ can be expressed using Equation (3). The area-weighted coefficients are computed using Equation (4) with the oriented triangle area method, where the triangle areas are taken as oriented areas.

(3) $$p^{\prime }=\lambda_{1}t_{1}+\lambda_{2}t_{2}+\lambda_{3}t_{3}$$

(4) $$\lambda_{1}=\frac{S(\Delta P^{\prime }T_{2}T_{3})}{S(\Delta T_{1}T_{2}T_{3})},\lambda_{2}=\frac{S(\Delta T_{1}P^{\prime }T_{3})}{S(\Delta T_{1}T_{2}T_{3})},\lambda_{3}=\frac{S(\Delta T_{1}T_{2}P^{\prime })}{S(\Delta T_{1}T_{2}T_{3})}$$

$\lambda_{1},\lambda_{2\;}and\;\lambda_{3}$ must satisfy Equation (5). If not, the ray does not intersect the triangle. In addition, if $\lambda_{1}$ > 0, $\lambda_{2}$ > 0, and $\lambda_{3}$ > 0, then the intersection point $P^{\prime }$ lies inside the triangular element or on its boundary. Otherwise, the intersection lies outside the triangular element and is not counted as a valid intersection.

(5) $$\lambda_{1}+\lambda_{2}+\lambda_{3}=1$$

(4) Determination of whether the intersection lies on the ray

The normal vector $h_{2}$ of the triangular element can be computed using Equation (6). Then, substituting it into Equation (7) to obtain the ray parameter n, and determine whether the intersection point $P^{\prime }\;$ lies on the ray (not on its opposite extension).

(6) $$h_{2}=e_{1}\times e_{2}$$

(7) $$n={(t}_{1}-p)h_{2}$$

If n > 0, then $P^{\prime }$ lies on the ray and is counted as one valid intersection. Otherwise, $P^{\prime }$ lies on the opposite extension of the ray and is not counted as a valid intersection.

It should be noted that inspection cells located at the ballast–polyurethane interface are prone to misclassification of material properties due to the centroid position. For example, a cell that partly lies within ballast but whose centroid falls in the polyurethane region may be misclassified as polyurethane. Therefore, mesh size optimization in Section 3 is employed to ensure a sufficiently fine inspection-cell size, thereby reducing interface misclassification and ensuring the reliability of material classification.

#### 2.2.2. Material Property Assignment and Model Construction

Based on the material classification results of the inspection cells via the virtual ray casting method, this section constructs a mesoscopic finite element model of the polyurethane-solidified ballast bed by means of standardized data recording and mapping onto the finite element mesh, thereby providing a high-accuracy numerical basis for subsequent mechanical response analyses.

**Figure 6:**
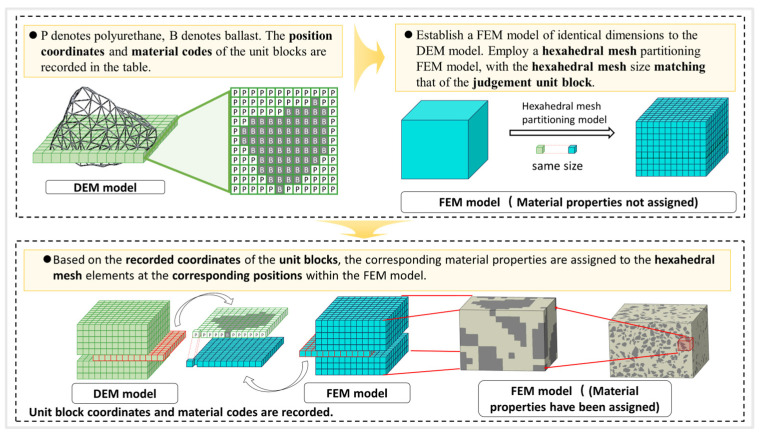
Flowchart of material property assignment and model construction. (In the figure, P denotes polyurethane and b denotes ballast; green cuboids represent the DEM model, blue cuboids represent the FEM model with unassigned material properties, and black-and-white mixed cuboids represent the FEM model with assigned material properties where white stands for polyurethane and black for ballast. The orange arrows indicate the modeling sequence.)

As shown in Figure 6, the finite element model is reconstructed in three steps: standardized data recording, same-scale mesh discretization, and material-property matching on the finite element mesh. The detailed procedure is as follows:

(1) Standardized data recording. To enable precise matching between each inspection cell and the subsequent finite element mesh, the core information of every inspection cell is recorded in a structured manner. The letter P (polyurethane) is used as the material code for the polyurethane phase, and B (ballast) as the material code for the ballast phase. A tabulated dataset is established containing the cell’s unique identifier, three-dimensional coordinates (x, y, z), and material code. The three-dimensional coordinates correspond exactly to the centroid of the inspection cell, ensuring full consistency with the spatial coordinate system of the discrete element model. The unique identifier prevents data confusion during subsequent mesh assignment.
(2) Same-scale mesh discretization. A rectangular cuboid with dimensions identical to those of the discrete element ballast model is constructed. It serves as the geometric basis of the finite element model. This cuboid is discretized with a structured hexahedral mesh, and the mesh size is set to strictly match the size of the inspection cells defined in Section 2.2.1 to enforce a one-to-one correspondence between each finite element mesh element and a single inspection cell, thereby avoiding material-property mis-mapping caused by any size mismatch between the mesh and the inspection cells.
(3) Material-property matching on the finite element mesh. The standardized dataset is imported, and material properties are automatically assigned according to a coordinate-matching rule: when a finite element’s centroid coordinate matches that of a “B” inspection cell, the element is assigned ballast parameters; when it matches a “P” inspection cell, the element is assigned polyurethane parameters.

The reconstructed finite element model fully preserves the irregular geometry and spatial distribution of the ballast. The polyurethane phase in the model is accurately represented as filling the inter-particle voids, thereby overcoming the limitation of conventional macroscopically homogenized models in capturing mesoscopic heterogeneity.

Based on the reconstruction result, the cross-material applicability of the proposed framework can be summarized as follows: (1) it is directly applicable to particulate–binder composites where a rigid particle skeleton can be reconstructed and the binder can be treated as a continuous phase filling the inter-particle voids, typical examples include polymer granular composites and coarse-scale asphalt mixture representations (coarse aggregates versus an equivalent asphalt mastic); (2) for materials that intrinsically require explicit multi-phase descriptions (e.g., concrete with mortar and interfacial transition zones, or fiber-reinforced composites), additional phase definitions and reconstruction strategies, together with dedicated validation, are necessary.

## 3. Determination of Mesh Size and Experimental Validation

Building on the modeling framework introduced in Section 2, this section focuses on selecting and validating the key parameter—the mesh size. A series of mesh sizes scaled by the minimum particle size is considered to evaluate the trade-offs among geometric fidelity, material-phase volume fractions, and computational cost, followed by a mesh-sensitivity assessment. The model responses are then benchmarked against laboratory test results to verify accuracy and reproducibility, thereby laying the foundation for subsequent parametric studies and engineering applications.

### 3.1. Effect of Mesh Resolution on Simulation Results

Mesh discretization is fundamental to building a finite element model. A smaller mesh size yields more elements, higher geometric fidelity, and results that are closer to reality. However, accuracy gains come with increased computational cost. Therefore, selecting an appropriate mesh size to balance cost and accuracy is essential.

In this study, a hexahedral mesh is used. The mesh size is expressed as a multiple of the minimum particle size *D*_min_ (10 mm in this study) for the ballast grading. A smaller multiplier before *D*_min_ corresponds to a smaller mesh size and higher resolution. Five mesh sizes are considered to span the range from high accuracy to high computational efficiency, facilitating mesh-resolution verification and strict alignment with geometric layering to achieve a balance between computational cost and accuracy. The corresponding numbers of finite element model elements for each size are given in Table 4.

**Figure 7:**
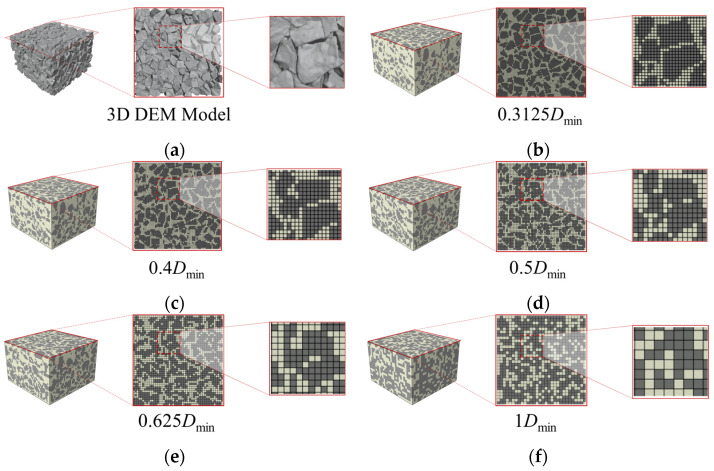
Comparison of finite element reconstructions of the same discrete element model using different mesh sizes; (**a**) Original 3D discrete element model; (**b**) Finite element model reconstructed with a mesh size of 0.3125 *D*_min_; (**c**) Finite element model reconstructed with a mesh size of 0.4 *D*_min_; (**d**) Finite element model reconstructed with a mesh size of 0.5 *D*_min_; (**e**) Finite element model reconstructed with a mesh size of 0.625 *D*_min_; (**f**) Finite element model reconstructed with a mesh size of 1 *D*_min_.

A comparison of models with different mesh sizes is shown in Figure 7. As the mesh is refined (smaller element size), the angularity and shape of the ballast particles are captured more faithfully, and the boundaries between adjacent particles become clearer.

As noted in Section 2, inspection cells located at the ballast–polyurethane interface are prone to classification errors, which can bias the estimated relative contents of ballast and polyurethane. Accordingly, the ballast volume fraction in the models under the different computational scenarios are analyzed.

**Figure 8:**
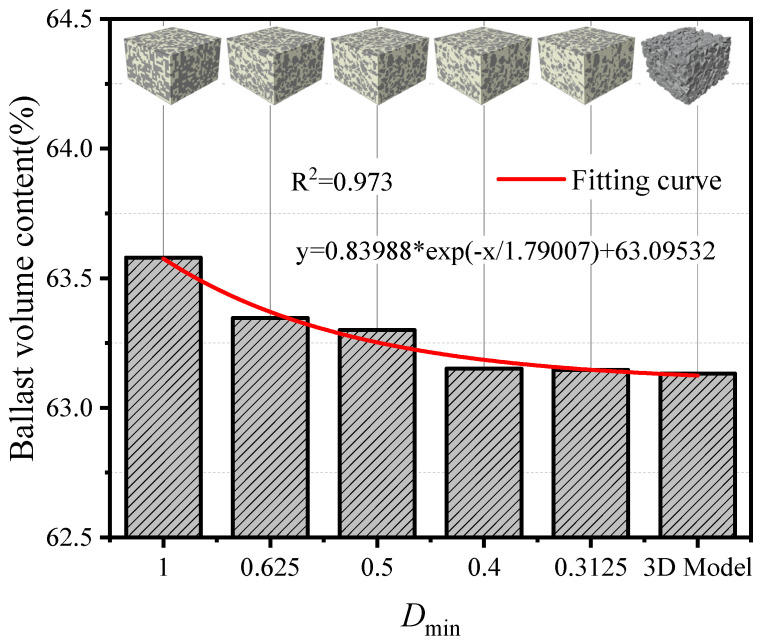
Comparison of ballast volume fraction between the original 3D discrete element model and the reconstructed finite element models at different mesh sizes.

Figure 8 compares the ballast volume fraction of the three-dimensional mesoscopic models at different mesh sizes with that of the three-dimensional discrete element model. It should be noted that, some ballast particles located on the model surface extend beyond the 300 mm × 300  mm × 200 mm domain. When computing its volume, these out-of-domain portions were subtracted. The results show that, as the mesh is refined (smaller element size), the ballast volume fraction in the mesoscopic model decreases approximately exponentially. When the mesh size reaches 0.4 *D*_min_, the decreasing trend weakens and the mesoscopic estimate approaches that of the three-dimensional model. This behavior occurs because ballast occupies a large share of the specimen volume. When the total number of inspection cells is limited, their centroids are more likely to fall within the ballast phase, leading to a relatively larger estimated ballast volume fraction for coarser meshes—the larger the mesh size, the larger the error.

### 3.2. Calibration of PU Material

Consistent with prior studies [59,60,61], this paper employs the generalized Maxwell constitutive model to characterize the mechanical behavior of the polyurethane material.

For a generalized Maxwell model comprising n spring–dashpot elements, the relaxation modulus can be expressed as:

(8) $$E(t)=E_{\infty }+\sum_{i=1}^{n}E_{i}\exp(-t/\tau_{i})=E_{0}\left(1-\sum_{i=1}^{n}e_{i}\left[1-\exp(-t/\tau_{i})\right]\right)$$

where *t* denotes time; $\tau_{i}$ is the relaxation time, $\tau_{i}=\eta_{i}/E_{i}$, $E_{i}$ is the relaxation modulus associated with the i-th Maxwell branch, $\eta_{i}$ is the dashpot viscosity of the i-th Maxwell branch; $E_{\infty }$ is the long-term (equilibrium) modulus; and $E_{0}$ is the instantaneous modulus; $e_{i}$ is the relative relaxation modulus of the i-th branch with respect to $E_{0}$, defined as $e_{i}=E_{i}/E_{0}$.

In three dimensions, the constitutive relations of the generalized Maxwell model can be written as:

(9) $$\sigma (t)=\int_{0}^{t}2G(t-s)\frac{de}{ds}ds+I\int_{0}^{t}K(t-s)\frac{d\phi }{ds}ds$$

Here ***σ***(*t*) is the stress as a function of time; *t* denotes the current time, and $s\in \left[0,t\right]$ is a dummy history-time (integration) variable; (*t* − *s*) is the time lag; e and ϕ are the mechanical deviatoric and volumetric strains; K is the bulk modulus and G is the shear modulus.

The relaxation functions *K*(*t*) and *G*(*t*) can be defined individually in terms of a series of exponentials known as the Prony series:

(10) $$G(t)=G_{0}(1-\sum_{i=1}^{n_{G}}g_{i}\left[1-\exp(-t/\tau_{i}^{G})\right])$$

(11) $$K(t)=K_{0}(1-\sum_{i=1}^{n_{K}}k_{i}\left[1-\exp(-t/\tau_{i}^{K})\right])$$

where $G_{0}$ denotes the instantaneous shear relaxation modulus; $\tau_{i}^{G}$ is the relaxation time of the i-th branch, i.e., the time required for the viscous resisting force in that branch to decay to $\exp\left(-1\right)$ of its initial value; and $g_{i}$ is the relative shear modulus of the i-th branch with respect to $G_{0}$, defined as $g_{i}=G_{i}/G_{0}$; $K_{0}$ denotes the instantaneous bulk modulus; $\tau_{i}^{K}$ is the relaxation time associated with the i-th bulk branch; and $k_{i}$ is the relative bulk modulus of the i-th branch with respect to $K_{0}$, defined as $k_{i}=K_{i}/K_{0}$.

The number of terms in bulk and shear, $n_{K}$ and $n_{G}$, need not equal each other. In fact, in many practical cases it can be assumed that $n_{K}=0$. Hence, the differential equation for the deviatoric stress tensor **S** characterizing material stress σ is:

(12) $$S=2\left(G_{\infty }e(t)+\sum_{i=1}^{n_{G}}G_{i}\int_{0}^{t}\exp(\left[(s-t)/\tau_{i}\right]\frac{de(s)}{ds}ds\right)$$

here **S** is the deviatoric stress tensor; $\tau_{i}$ is the relaxation time.

Where the viscous (creep) strain in each term of the series is defined as:

(13) $$e_{i}\left(t\right)=\int_{0}^{t}\left(1-\exp\left[(s-t)/\tau_{i}\right]\right)\frac{de\left(s\right)}{ds}ds$$

where $e_{i}$ denotes the deviatoric strain internal variable associated with the i-th branch, and e denotes the deviatoric strain tensor.

By substituting Equation (13) into Equation (12), Equation (12) can be rewritten as:

(14) $$S=2G_{0}\left(e\left(t\right)-\sum_{i=1}^{n}g_{i}e_{i}\left(t\right)\right)$$

**Figure 9:**
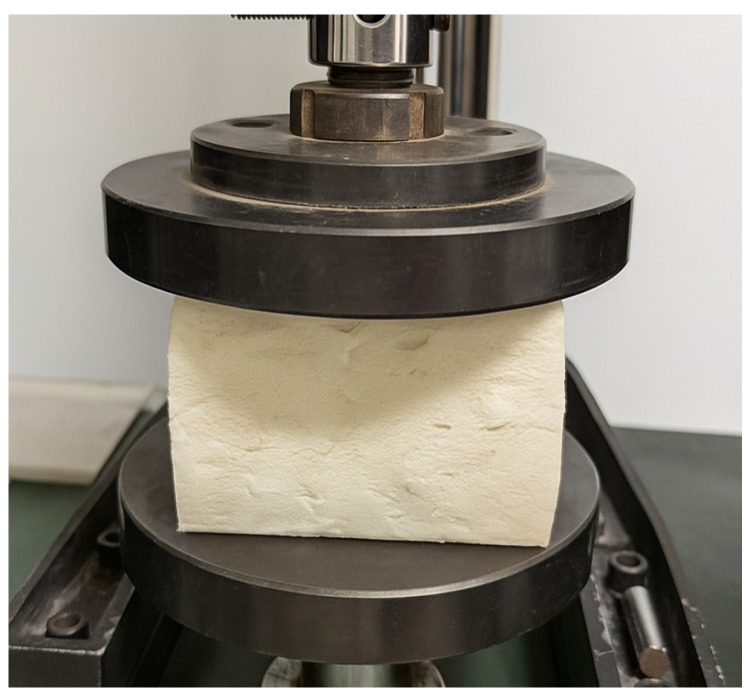
The stress relaxation experiment for polyurethane material.

It can be observed that the key to accurately calibrating the parameters of polyurethane material lies in determining the Prony series parameters $g_{i}$ and $\tau_{i}$. Therefore, in this study, a stress relaxation experiment for polyurethane material is designed. The polyurethane specimen has dimensions of 100 mm × 100 mm × 80 mm and a density of 160 kg/m^3^. The specific experimental procedure is as follows: (1) The polyurethane specimen is placed between two steel plates, with the upper plate applying load and the lower plate fixed; (2) A sudden load is applied to induce a certain amount of deformation in the specimen, which is then maintained constant, while time and force are continuously recorded; (3) The acquired data is imported into a program for analysis to determine the values of $g_{i}$ and $\tau_{i}$. The stress relaxation experiment for polyurethane material is shown in Figure 9, and the obtained Prony series parameters are listed in Table 5.

**Figure 10:**
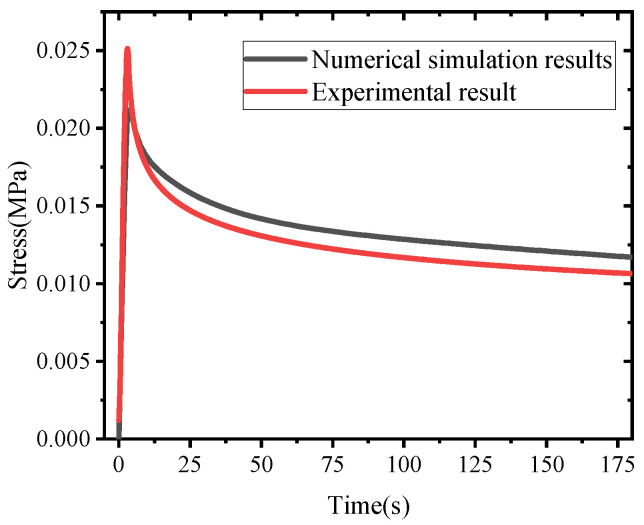
Comparison of Experimental and Numerical Simulated Stress-Time Curves in Stress Relaxation Test.

A finite element relaxation simulation was conducted using the calibrated Prony-series parameters, following the same strain-hold condition as the laboratory stress relaxation test. As shown in Figure 10, the numerical stress–time curve agrees well with the experimental result over the investigated time window, indicating that the calibrated parameters can reliably capture the time-dependent relaxation behavior of the cured polyurethane.

In this study, the parameters of the ballast were determined based on reference [62]. For polyurethane, our previous study [26] indicate that the instantaneous modulus exhibits an approximately linear elastic response within the deformation range considered. Therefore, the polyurethane phase is described using a combined linear elastic and viscoelastic constitutive formulation. The elastic parameters of both ballast and polyurethane are listed in Table 6.

### 3.3. Laboratory Test

Uniaxial compression was selected in this study as the validation case, mainly for the following reasons. In railway engineering, when loads are transmitted from the sleeper to the ballast bed, vertical compression is the predominant loading mode. Meanwhile, uniaxial compression provides a direct and quantifiable benchmark for mesh-sensitivity analysis and for evaluating the load-transfer response under compressive loading. Therefore, this study focuses on the uniaxial compression scenario and does not investigate the behavior of polyurethane under other loading modes.

To calibrate the material parameters of the numerical model and determine an appropriate mesh size, specimens were fabricated using the same gradation as in the simulations, and laboratory tests were conducted.

**Figure 11:**
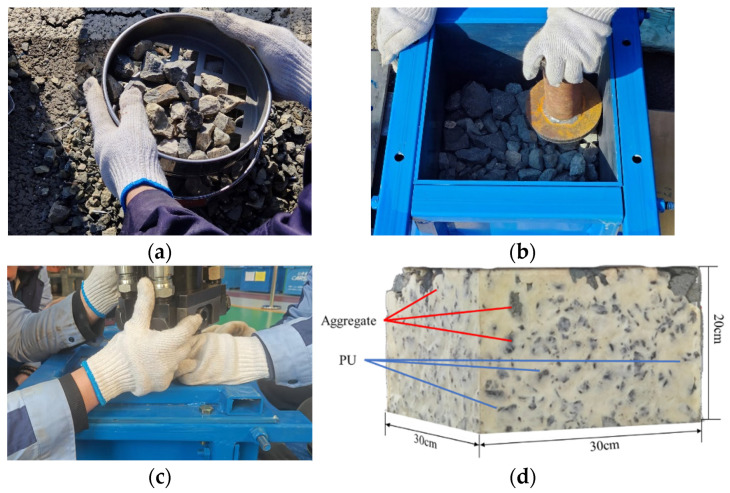
Test Specimen Fabrication Procedure; (**a**) Sieving and weighing of ballast; (**b**) Layer-by-layer placement of ballast; (**c**) Pouring polyurethane; (**d**) Polyurethane-solidified ballast bed specimen.

**Figure 12:**
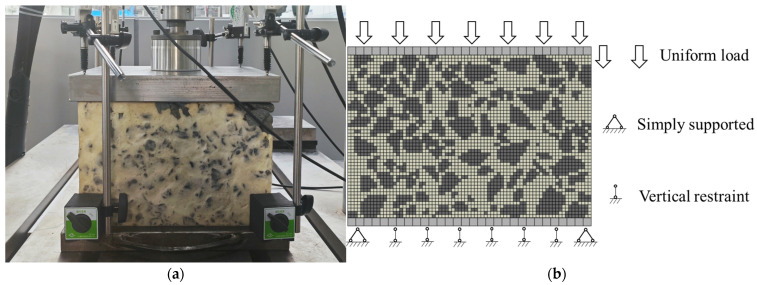
Schematic of the numerical simulation and the laboratory test; (**a**) Laboratory uniaxial compression test; (**b**) Loading and boundary conditions for the finite element simulation.

As shown in Figure 11, the fabrication procedure for the polyurethane-solidified ballast bed specimens was as follows: (1) preparing a steel mold measuring 30 cm × 30 cm × 20 cm, with five holes on the top cover for polyurethane casting; (2) sieving and weighing the ballast to achieve the prescribed gradation; (3) applying a static load to compact the ballast; (4) sealing the mold; (5) pouring the polyurethane; (6) demolding after a 6 h rest. In the laboratory uniaxial compression test, a loading-plate was used, and the load was increased at a constant rate and uniformly transferred to the specimen until the target load of 30 kN was reached. The laboratory setup is illustrated in Figure 12a.

A uniaxial compression test was simulated for the polyurethane-solidified ballast bed specimen. The specimen dimensions and gradation were identical to those in the laboratory test. The external load was applied to the specimen through an upper rigid plate, acting perpendicular to the x–y plane, and was increased at a constant rate until reaching the target load of 30 kN. The lower rigid plate was fixed, while the upper rigid plate bore and transmitted the load. A friction coefficient of 0.2 was used at the interfaces between the specimen and the rigid plates. The loading and boundary conditions of the simulation are shown in Figure 12b.

### 3.4. Results Analysis

**Figure 13:**
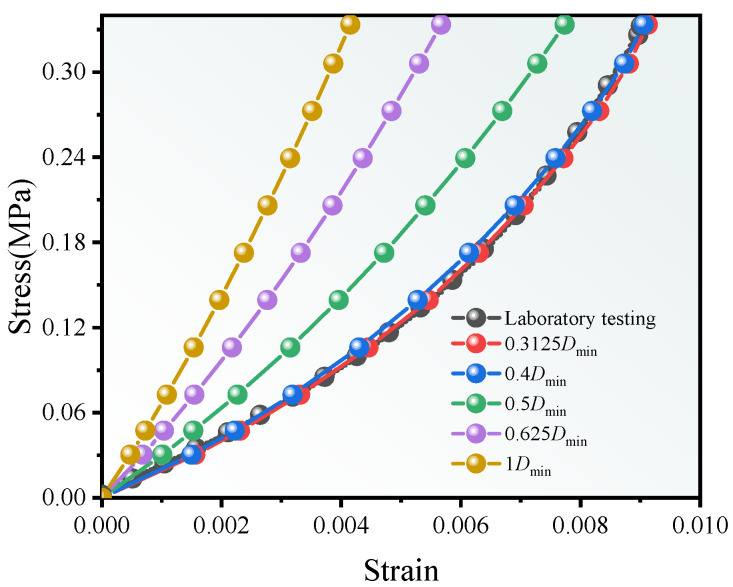
Stress–strain responses from numerical simulation and laboratory test.

A validation comparison between the numerical stress–strain responses under different mesh-size cases and the laboratory test is shown in Figure 13. The results indicate that, when the mesh size is greater than 0.4 *D*_min_, a smaller mesh yields a smaller strain at the same stress level. When the mesh size is ≤0.4 *D*_min_, the influence of mesh size on the results can be ignored and the simulation results approach the laboratory measurements, indicating high model accuracy at this resolution.

**Figure 14:**
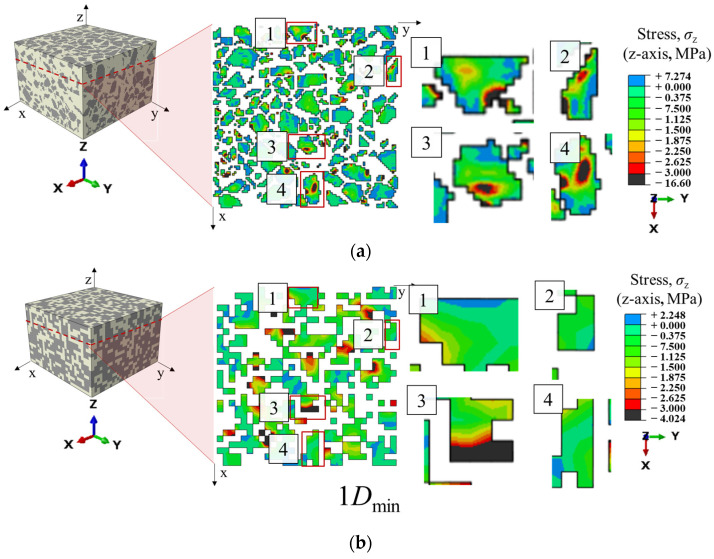
Comparison of z-direction stress contour maps of ballast particles on the cross-section at the same location for the coarsest (1 *D*_min_) and finest (0.3125 *D*_min_) mesh sizes (**a**) Cross-sectional stress contour of the finite element model with a mesh size of 0.3125 *D*_min_; (**b**) Cross-sectional stress contour of the finite element model with a mesh size of 1 *D*_min_.

Figure 14 compares the contour maps of the stress along z direction of the ballast particles at a cross section of the model for the mesh sizes of 1 *D*_min_ and 0.3125 *D*_min_. When the mesh size is 1 *D*_min_, the boundaries between ballast particles are blurred and many particles fuse into an apparent continuum. Relative movement between particles is inhibited, which affects the overall deformation of the model. When the mesh size is 0.3125 *D*_min_, particles with more realistic shapes can move relative to each other, and the global deformation is closer to reality. Therefore, as the mesh is refined, the model exhibits larger strain at the same stress level.

In summary, when the mesh size is less than or equal to 0.4 *D*_min_, the model reproduces the angularity and shape of ballast particles well and delineates particle–particle contact boundaries clearly. Meanwhile, the influence of mesh size on the results becomes negligible, and the numerical simulations agree closely with the laboratory tests, indicating high model accuracy. Considering the trade-off between computational cost and accuracy, a mesh size of 0.4 *D*_min_ is adopted in this study.

## 4. Case Study: Effect of Sleeper Width on the Mechanical Response of the Ballast Bed

The sleeper is a key component of the track with the polyurethane-solidified ballast bed. In prior practice it has largely been designed as an isolated track component, with limited coordinated design between sleeper and the overall track structure. Because the track functions as an integrated system, the design of sleeper parameters should not only satisfy load-bearing strength requirements but also account for their influence on the global performance of the track so as to reduce maintenance demand. Accordingly, this section examines the mechanical response of the ballast bed and analyzes the effect of sleeper width, providing guidance for the coordinated design of track structures with the polyurethane-solidified ballast.

### 4.1. Design of Computational Scenarios

#### 4.1.1. Discussion of Issues

The external load on the track is transmitted from the sleeper into the ballast bed. Within the ballast bed, it diffuses from an initially localized state outward and downward. The more fully the diffusion develops and the wider its coverage, the more uniform the load transfer, and the greater the ballast bed’s overall load-bearing and deformation-control capacity. Conversely, insufficient diffusion produces stress concentration and elevated peaks near the contact region, hindering the ballast bed from acting as an integral system. On this basis, this section focuses on the stress-diffusion inside the ballast bed and the influence of sleeper width.

**Figure 15:**
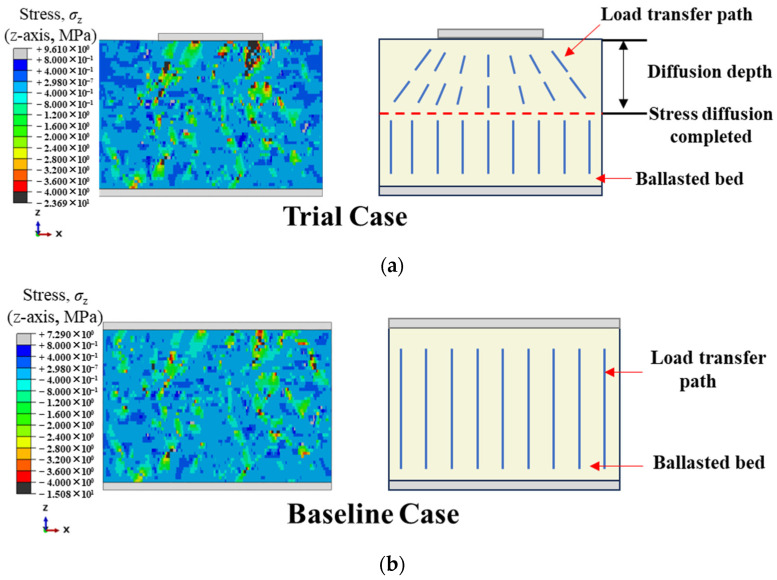
Schematic illustration of the load transfer path in the Trial Case and the Baseline Case. (**a**) Stress load transfer path in the Trial Case; (**b**) Stress load transfer path in the Baseline Case.

To this end, two cases were configured following the modeling method described above and evaluated by numerical trial calculations. As shown in Figure 15, in the Trial Case, a top loading plate is used to simulate the sleeper, and the sleeper does not fully cover the ballast-bed top surface. In the Baseline Case, the sleeper bottom surface fully coincides with the ballast-bed top surface in the projection onto the x–y plane. The same external load is applied in both cases.

The load transfer path in Figure 15 shows clear differences between the Trial Case and the Baseline Case. In the Trial Case, the load propagates downward along the z-axis from the sleeper–ballast contact region and gradually evolves from a localized state to stress diffusion within the x–y section. In the Baseline Case, because the sleeper bottom fully coincides with the ballast-bed top in the x–y projection, the stress is uniformly transmitted across the entire section from the onset of loading. It serves as the benchmark for the completed-diffusion state. To quantify the diffusion in the Trial Case, the diffusion depth is defined as the vertical distance from the sleeper bottom to the first horizontal section at which the stress has expanded from the contact region to the entire section and the load state on that x–y section is essentially consistent with that of the Baseline Case at the same location.

**Figure 16:**
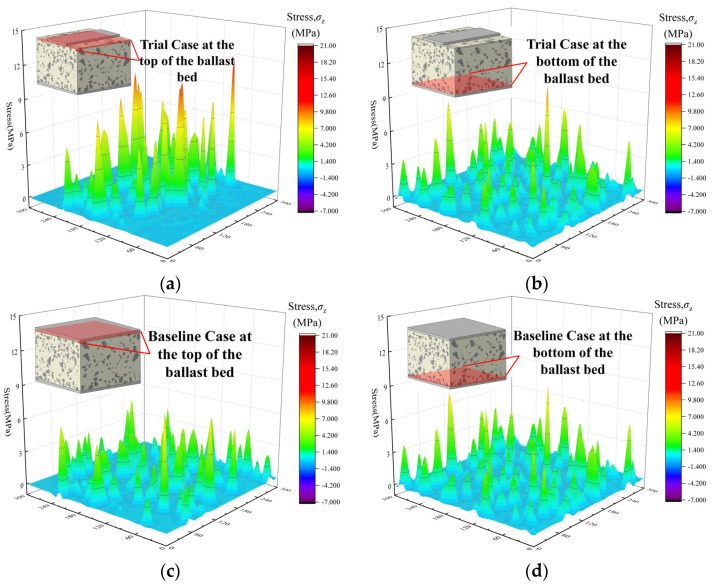
Three-dimensional cross-sectional stress distributions of the ballast bed for the two cases; (**a**) Top surface of the ballast bed (Trial Case); (**b**) Bottom surface of the ballast bed (Trial Case); (**c**) Top surface of the ballast bed (Baseline Case); (**d**) Bottom surface of the ballast bed (Baseline Case).

Figure 16 presents the stress distributions on the top and bottom surfaces of the ballast bed for the two cases. At the top surface, stress diffusion has not yet been completed in the Trial Case, and the stress is concentrated in the sleeper–ballast contact region. In contrast, the top-surface stress distribution is relatively uniform in the Baseline Case because the sleeper bottom fully coincides with the ballast-bed top surface in the x–y projection. With increasing depth to the bottom surface where stress diffusion is completed, the stress in the Trial Case has expanded from the contact region to the entire section, and the stress distribution becomes essentially consistent with that of the Baseline Case. This indicates that, under the same external load, the internal responses of the two cases are comparable at the completed-diffusion location, confirming that the current loading scheme is reasonable. Accordingly, the Baseline Case serves as the benchmark for completed diffusion. When the stress distribution on an x–y section at any height matches that of the Baseline Case at the same location, stress diffusion is deemed to be completed there.

**Figure 17:**
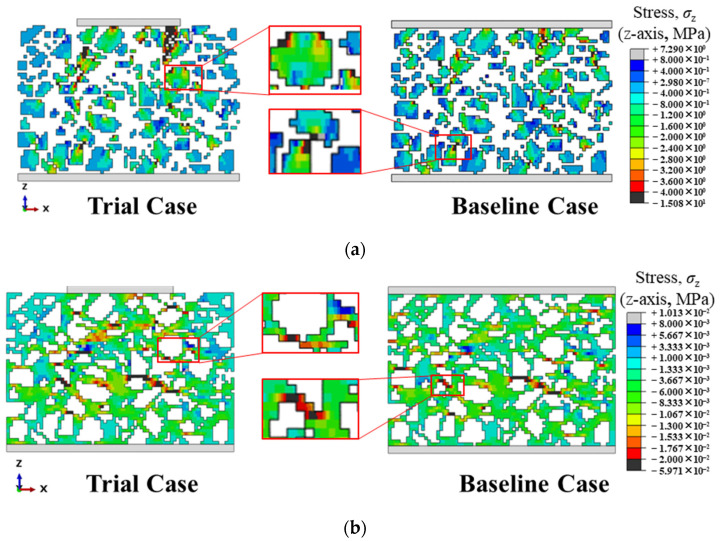
Single-phase stress contour maps: ballast phase and polyurethane phase; (**a**) Ballast phase; (**b**) Polyurethane phase.

To present the mechanical characteristics of each material more clearly, the stress contour maps of the ballast and polyurethane are displayed separately in Figure 17. As shown in Figure 17a, stress concentrations occur mainly at the angular corners of particles within contact regions between adjacent particles. Figure 17b indicates that the polyurethane filling the inter-particle voids acts as a buffer. When the polyurethane layer interposed between two ballast particles is insufficiently thick, local stress concentration is more likely to develop. Compared with the conventional DEM, the present numerical model not only identifies force chains but also directly resolves the intra-particle stress distribution within ballast. Whereas traditional DEM typically approximates the polyurethane phase with bond models, here the polyurethane filling between ballast particles is simulated explicitly by solid elements, which better reflects actual conditions and clearly reveals the stress and deformation characteristics of the polyurethane.

#### 4.1.2. Case Definitions

**Figure 18:**
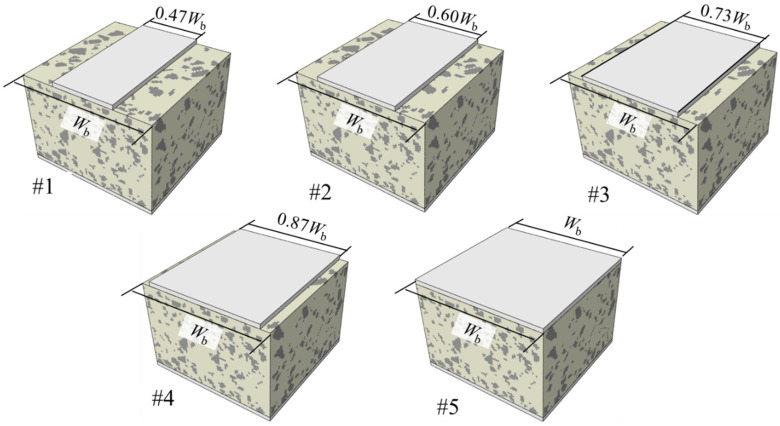
Schematic finite element models for each case.

Five cases were designed for analysis. The case configurations are shown in Table 7. In Case #5, the sleeper bottom surface fully coincides with the ballast-bed top surface, serving as the Baseline Case. In the table, the ballast-bed width *W*_b_ is 300 mm, and the applied load represents a heavy-haul line condition. Schematic finite element models for each case are shown in Figure 18.

**Figure 19:**
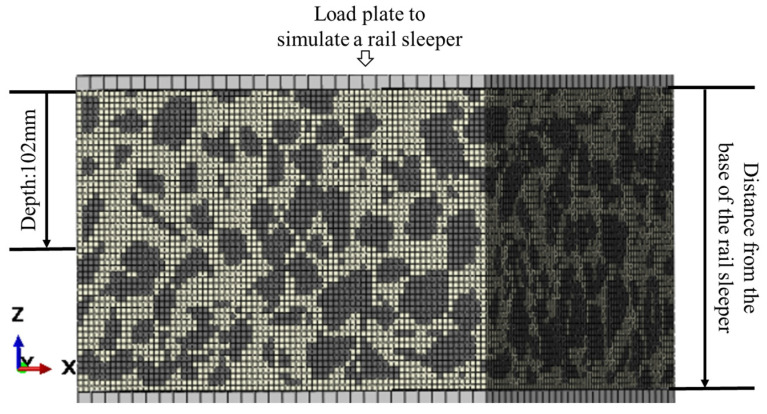
Coordinate system of the finite element model.

As shown in Figure 19, the specimen is divided into 50 layers with a layer thickness of 4 mm. The vertical distance from the sleeper bottom to the centroid of each layer is defined as the depth. For clarity, a Cartesian coordinate system is adopted to describe the orientation of the finite element model.

### 4.2. Results and Discussion

#### 4.2.1. Stress Response

**Figure 20:**
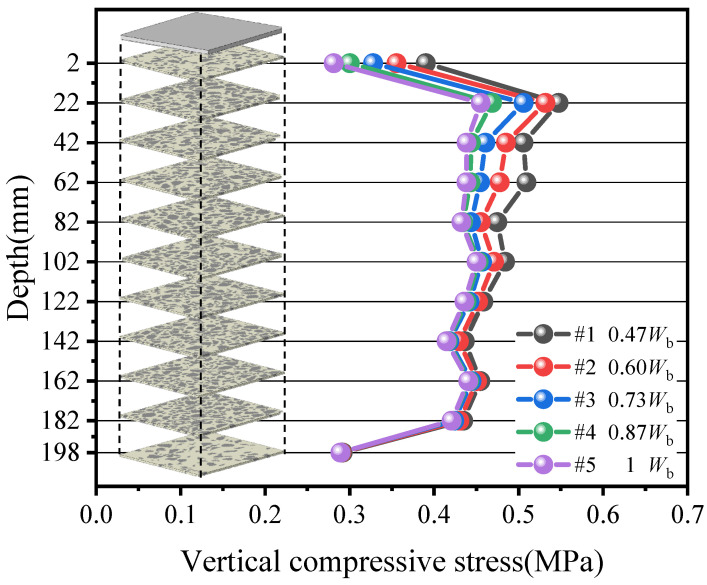
Vertical compressive stress on planes at different depths.

To reveal the stress diffusion inside the specimen, the vertical compressive stress of each layer at different depths was extracted and plotted as a line-and-marker chart, as shown in Figure 20. The results indicate that near the bottom of the sleeper, stress diffusion is not yet completed and the vertical compressive stress is high. The compressive stress decreases and progressively diffuses with increasing depth until it approaches a stable level beyond a certain depth. It is also observed that the compressive stress at the top and bottom surfaces of the ballast bed is overall smaller. This can be attributed to two factors. Firstly, due to ballast particle angularity and geometric constraints from the box walls, the top and bottom layers contain a higher proportion of polyurethane and a lower proportion of ballast. Secondly, the top and bottom contact surfaces are relatively smooth, which limits sharp ballast–ballast contacts and force-chain concentration, resulting in a lower average compressive stress.

To facilitate a comparison of the effect of sleeper width on stress diffusion, the following criterion is adopted to determine a diffusion depth. Taking Case #5 as the Baseline Case, stress diffusion is deemed completed when the vertical compressive stress at a cross-section of the diffusion depth for any case exhibits a relative difference of no more than 5% with respect to Case #5. Moreover, at that depth and at all greater depths, the relative difference with Case #5 continues to remain within 5% to ensure distribution uniformity and comparability among cases.

The relative difference Δ of stress is computed in Equation (15).

(15) $$\Delta =\frac{\left(\sigma_{i}-\sigma_{\#5}\right)}{\sigma_{\#5}}\times 100\%(i=\#1,\#2,\#3,\#4)$$

**Figure 21:**
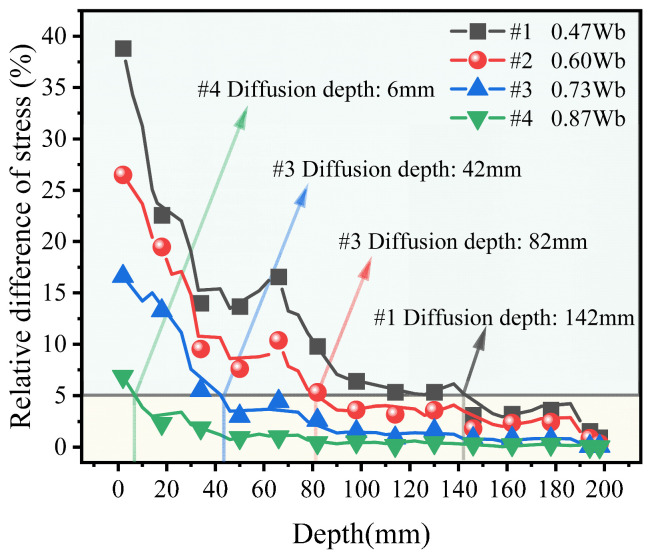
Relative difference in stress–depth curve.

The calculation results are shown in Figure 21. The diffusion depth increases as the sleeper becomes narrower. Specifically, the diffusion depth is about 6 mm for Case #4 (0.87 *W*_b_), about 42 mm for Case #3 (0.73 *W*_b_), about 82 mm for Case #2 (0.60 *W*_b_), and about 142 mm for Case #1 (0.47 *W*_b_), respectively.

**Figure 22:**
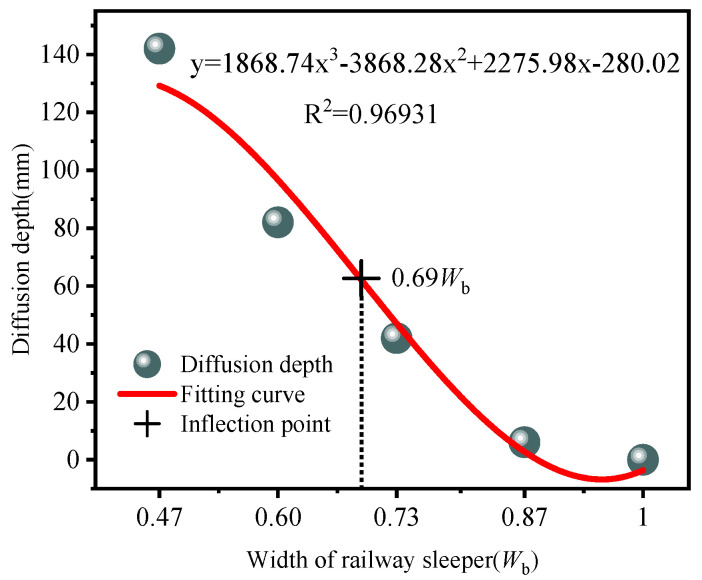
Fitted curve of diffusion depth for the cases.

The diffusion depth for all cases and a fitting curve are plotted in Figure 22. reveals an inflection point at about 0.69 *W*_b_, close to Case #3 (0.73 *W*_b_). For sleeper widths below this inflection, the curve is steep, and each further reduction in width sharply increases diffusion depth, indicating a marked decline in stress diffusion capacity; above the inflection, the curve gradually flattens. Accordingly, from the standpoint of stress diffusion performance, a sleeper width not less than 0.73 *W*_b_ is recommended.

#### 4.2.2. Displacement Response

**Figure 23:**
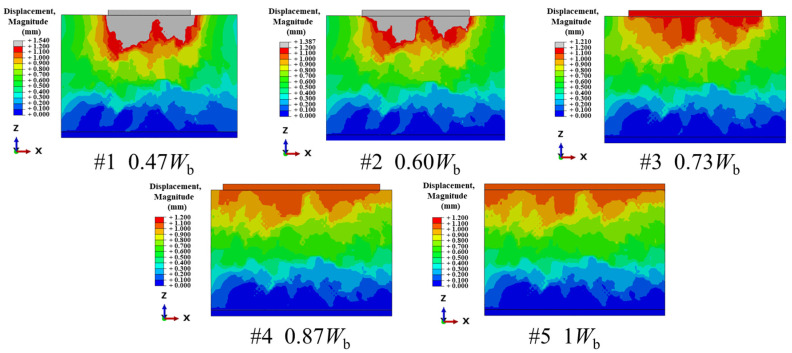
Displacement contour maps for the cases.

Figure 23 shows the contour maps of displacement for different sleeper widths. The displacement is largest directly beneath the sleeper and decreases layer by layer with depth. Correspondingly, the differences among cases diminish with increasing depth. Laterally, a wider sleeper produces a more dispersed high-displacement zone and smoother isolines, indicating more sufficient diffusion of deformation within the ballast bed. When the sleeper is narrow (e.g., 0.47 *W*_b_–0.60 *W*_b_), the high-displacement zone is concentrated beneath the sleeper with a steep lateral gradient, and local displacement is more pronounced. Accordingly, to reduce sleeper displacement and promote more uniform transmission, a sufficient sleeper width should be designed.

**Figure 24:**
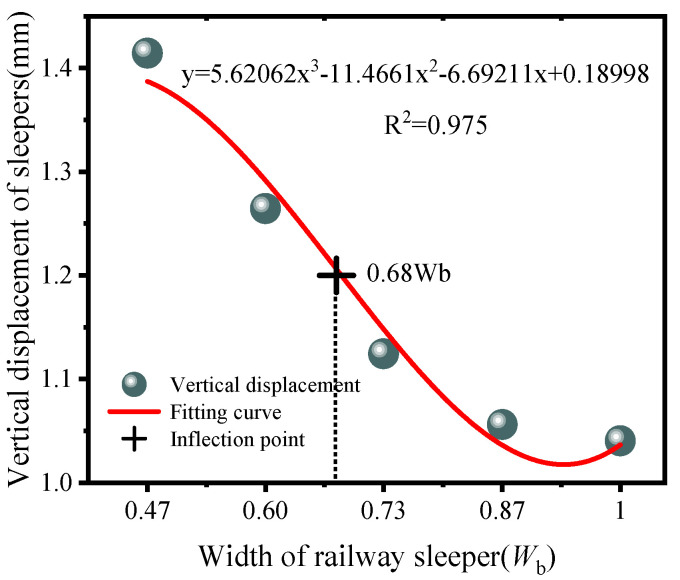
Fitted curve of sleeper displacement for the cases.

Figure 24 shows the vertical displacement of the sleeper under different cases. The numerical results indicate that, at the same load level, a smaller sleeper width leads to a larger displacement. A cubic fit suggests that the displacement–width relationship can be represented by a third-order polynomial, with an inflection abscissa at about 0.68 *W*_b_. Using this inflection as a threshold, when the sleeper width is greater than 0.73 *W*_b_, reducing the width causes only a slow increase in displacement; when the sleeper width is less than 0.73 *W*_b_, the displacement becomes more sensitive to width, and further reduction triggers a rapid increase.

This displacement response is consistent with the stress response: as sleeper width decreases, the diffusion depth becomes longer and stress diffusion efficiency decreases, leading to local stress concentration in the polyurethane-solidified ballast bed and larger local displacements of both the sleeper and the ballast. In Figure 22, the fitted diffusion-depth–width curve shows an inflection at 0.69 *W*_b_, while in Figure 24, the fitted displacement–width curve shows an inflection at 0.68 *W*_b_. Therefore, considering both diffusion depth and displacement control, a sleeper width not less than 0.73 *W*_b_ is recommended.

## 5. Conclusions

The polyurethane-solidified ballast bed is a typical heterogeneous material formed by injecting a polyurethane ballast binder into a conventional granular ballast matrix so that the binder fills the inter-particle voids. Owing to the material complexity of the polyurethane-solidified ballast bed, numerical studies based solely on the discrete element method (DEM) cannot faithfully represent the mesoscopic actual state of the ballast bed. Digital image-based techniques used for mesoscopic modeling are constrained by specimen size and therefore cannot meet the research needs for this system. To address these limitations, this study integrates the DEM, the FEM, and a virtual ray casting-based approach for constructing a mesoscopic heterogeneous model of the composite, enabling high-fidelity simulation at the meso-scale for the polyurethane-solidified ballast bed. On this basis, designs with different sleeper widths are analyzed to evaluate their influence on the load–deformation characteristics of the polyurethane-solidified ballast bed, and an optimized sleeper width is proposed. The main conclusions are as follows:

(1) For the mesoscopic reconstruction of the heterogeneous polyurethane-solidified ballast bed, this study employs three-dimensional laser scanning to capture the geometry of ballast particles and generate a granular ballast bed that faithfully reflects particle irregularity. A virtual ray casting procedure is used to extract geometric information and identify the spatial locations of the ballast and polyurethane phases. A finite element model integrating both phases is established for numerical analysis. Comparison with laboratory tests demonstrates good accuracy of the model. The proposed framework is applicable not only to polyurethane-solidified ballast beds but also to other multi-material composites. Its extension is mainly suitable for particulate–binder composites with a reconstructable particle skeleton. Multi-phase systems such as concrete (including mortar and interfacial transition zones) would require additional phase reconstruction and validation; nevertheless, with appropriate extensions, the proposed framework remains applicable.
(2) For the proposed modeling method, the selection of mesh size in the finite element model is critical to ensuring accuracy. Considering the fidelity of different mesh resolutions in reproducing ballast geometry and spatial distribution, their effectiveness in representing the stress field, and computational efficiency, the study indicates that a mesh size of 0.4 *D*_min_ satisfies the geometric accuracy requirements while keeping the total number of finite elements acceptable.
(3) From the perspective of the mesoscopic stress state of the polyurethane-solidified ballast bed, stress concentrations in the ballast occur mainly at the angular contact regions between neighboring particles. The polyurethane filling the inter-particle voids acts as a buffer, and when the polyurethane layer between two ballast particles is insufficiently thick, local stress concentration is more likely to develop. Compared with the conventional DEM, the numerical model built in this study not only identifies force chains but also directly resolves the intra-particle stress distribution within ballast particles.
(4) Due to boundary effects, the top and bottom of the polyurethane-solidified ballast bed exhibit a higher polyurethane volume fraction and a lower ballast fraction, resulting in reduced load-bearing capacity. These layers therefore constitute the weak zones of the system. The results further indicate that, under loading, the ballast serves as the primary load-carrying skeleton, whereas the polyurethane mainly cushions the load and resists the relative movement between ballast particles by providing interparticle bonding, thereby reducing plastic deformation induced by the relative movement of ballast particles.
(5) Sleeper width is a key factor governing the sleeper’s vertical displacement and the ballast-bed stress state under train loading. From the ballast-bed load transfer behavior, an inflection point occurs at a sleeper width of 0.69 *W*_b_. Below this point, the diffusion depth increases distinctly as width decreases, indicating a marked reduction in stress diffusion efficiency, whereas above the inflection the increase in diffusion depth becomes gradual. Overall, a sleeper width not less than about 0.73 *W*_b_ is recommended.

Future work will extend the present framework from monotonic loading to fatigue loading conditions. In particular, damage-related behaviors of polyurethane and the ballast–polyurethane interaction will be incorporated to evaluate how time-dependent viscoelasticity and damage accumulation influence stress diffusion, load transfer path, and long-term deformation of polyurethane-solidified ballast beds. In addition, curing-induced polyurethane density heterogeneity will be investigated, and spatially varying material properties will be introduced in the mesoscale model to evaluate their effects on the load transfer path and stress transfer characteristics.

## Figures and Tables

**Table 1:** Strengths and limitations of commonly used approaches for polyurethane-solidified ballast bed.

Modelling Methodology	Strengths	Limitations
Finite Element Method (FEM)	High engineering implementability; well-suited to large-scale and multi-scenario simulations.	Cannot faithfully capture the heterogeneity of the ballast–polyurethane composite or the aggregate-morphology-controlled local stress concentrations.
Discrete Element Method (DEM)	Explicitly resolves particle distributions and captures contact, sliding, rearrangement, and force-chain topology; polyurethane bonding can be represented by the bond, making the approach suitable for modeling contact-point consolidation.	Unable to accurately represent the intergranular pore-filling configuration characteristic of filling-type polyurethane-solidified ballast.
X-ray Computed Tomography (XCT) and Image Processing	High geometric fidelity with accurate mesostructural reconstruction.	Cannot accommodate engineering-scale polyurethane-solidified ballast specimens; moreover, XCT requires costly instrumentation and repeated specimen preparation, scanning, and image post-processing, incurring significant time and economic burdens.

**Table 2:** Ballast particle gradation.

Sieve Aperture (mm)	10	16	25	31.5	35	40
Percentage of sieved material (%)	0~5	16~30	50~60	70~90	90~100	100

**Table 3:** Comparative Synopsis of Mesoscale Modeling Methodologies.

Method	Random Aggregate Models (RAMs) [49,50,51,52,53,54]	Voronoi Tessellation Technique [55,56,57]	Mapping Interpolation Method [58]
Workflow	Treats concrete as a multiphase composite (aggregates, mortar, ITZs) in a representative volume element; simulates behavior using finite element methods.	Divides space into non-overlapping convex polyhedral cells via seed points; cells are shrunk/reshaped to generate irregular aggregates.	Maps material properties of aggregates, mortar, and ITZs to nodes of arbitrary mesh via nearest-neighbor interpolation.
Advantage	Captures mesoscale heterogeneity, predicts macroscopic properties and damage.	Auto-generates non-overlapping aggregates, high efficiency, enables high volume fraction.	Strong mesh adaptability, reduces mesh count, improves efficiency while maintaining accuracy.
Disadvantage	High computational cost, complex meshing, difficult parameter calibration.	Surfaces tend parallel to neighbors, limited geometric fidelity.	Lower accuracy in capturing localized stress concentrations vs. fine-mesh methods.
Highlight	Overcomes homogenization limitations, refined tool for failure analysis.	Eliminates interference detection, supports multi-phase modeling.	Resolves mesh density bottleneck, enables large-scale simulations.

**Table 4:** Number of elements for models with different mesh sizes.

Mesh Size	0.3125 *D*_min_	0.4 *D*_min_	0.5 *D*_min_	0.625 *D*_min_	1 *D*_min_
Number of elements	589,824	281,250	144,000	73,728	18,000

**Table 5:** Prony series parameters.

i	$\tau_{i}$	$g_{i}$
1	2.231	0.2559
2	21.5	0.2192
3	509.7	0.355

**Table 6:** Material Parameters Used in the Simulation.

Material	Density (kg/m^3^)	Modulus of Elasticity (MPa)	Poisson Ratio
ballast	2700	35,000	0.25
polyurethane	160	0.2336	0.14

**Table 7:** Case Identification and Description Table.

Case ID	Sleeper Width	Ballast-Bed Width	Load (kN)
#1	0.47 *W*_b_ (141 mm)	*W*_b_ (300 mm)	22.5
#2	0.60 *W*_b_ (180 mm)		
#3	0.73 *W*_b_ (219 mm)		
#4	0.87 *W*_b_ (261 mm)		
#5	1 *W*_b_ (300 mm)		

## Data Availability

The original contributions presented in this study are included in the article. Further inquiries can be directed to the corresponding author.

## Abbreviations

The following abbreviations are used in this manuscript:

XCT	X-ray computed tomography
DEM	Discrete Element Method
FEM	Finite Element Method
PSBB	polyurethane-solidified ballast beds
